# Depressive Disorders, Cognitive and Physical Function of Older People in Early Dementia Detection

**DOI:** 10.3390/life13102010

**Published:** 2023-10-04

**Authors:** Beata Pietrzak, Jolanta Kujawa, Anna Lipert

**Affiliations:** 1 Department of Medical Rehabilitation, Medical University of Lodz, 92-213 Lodz, Poland; beata.pietrzak@umed.lodz.pl (B.P.); jolanta.kujawa@umed.lodz.pl (J.K.); 2 Department of Sports Medicine, Medical University of Lodz, 92-213 Lodz, Poland

**Keywords:** depressive disorders, cognitive function, physical function, dementia, older people

## Abstract

Background: Aging is associated with cognitive decline, leading to cognitive and physical impairments, which are risk factors for loss of independence and dementia development. Early diagnosis is beneficial for both, the patient and their family, to avoid long-term consequences. The aim of this study was to analyze the frequency of depressive disorders and their influence on cognitive and physical function of older people in early dementia detection. Methods: There were 852 patients, aged at least 60 years, from the Central Teaching Hospital. The study was conducted between September 2022 and June 2023. The qualified participants were examined using four tools: Geriatric Depression Scale (GDS), Instrumental Activities of Daily Living (IADL), Timed Up and Go (TUG) and Schulman’s Clock-Drawing Test. Results: Over one-third had depressive disorders. A relationship with *p* < 0.05 was observed between GDS and IADL: r = −0.61. A relationship with *p* > 0.05 was observed between GDS and TUG: r = −024. A relationship with *p* < 0.05 was observed between GDS and CDT: r = 0.74. Conclusions: The first signs of depressive disorders in older people may be considered an indication for further diagnosis of dementia.

## 1. Introduction

Aging of societies is a global phenomenon, especially in highly developed countries [1]. Aging is associated with the deterioration of cognitive functions, leading to their impairment and dementia, which, together with the impairment of physical functions, leads to a loss of independence [2]. The search for effective solutions to prevent the loss of independence of the elderly by detecting emerging physical, mental and social dysfunctions as soon as possible is the theme of many studies [3]. The mutual relationship between the deterioration of cognitive functions, physical functions, mental functions and independence in everyday life should be emphasized.

Many reports emphasize that a common problem is the lack of screening for cognitive decline. Meanwhile, early diagnosis of dementia allows for avoiding long-term consequences [4], such as deterioration of thinking skills leading to loss of independence and deterioration of physical functions. It is important to note that cognitive impairment affects tens of millions of people worldwide, not only patients but also their caregivers, placing a financial burden on families and healthcare systems. It is interesting to study cognitive and physical functions at the same time, as both are associated with the risk of loss of independence and a higher risk of dementia [5]. The limited success of current treatments has led to the search for early markers of dementia that could predict future progression or improve the quality of life of people already suffering from the disease [6].

The risk of cognitive impairment increases with age due to physical disability [5,7]. This risk increases if it is accompanied by a noticeable decrease in walking speed [8]. In addition, a slower gait is associated with worse results in tests of attention, executive function, and memory [9]. Identifying early physical decline in older adults is important, but individuals who experience decline can usually ignore early changes in physical functioning and therefore do not report them [10]. The lack of adequate epidemiological studies on comprehensive measurements of physical fitness in the elderly also makes it difficult to compare functional status across countries and cultures [11,12]. Physical fitness is a key indicator of the health status of older people. According to gerontological studies, physical fitness may be an important predictor of later disability, significant morbidity and mortality, or may help predict future health needs and the use of social care [6]. The research also confirms the hypothesis that the loss of physical fitness may be an additional risk factor for the clinical onset of Alzheimer’s disease [7,13].

The deteriorating condition of the elderly leads to dependence, i.e., the inability to live independently and requires constant or long-term care and assistance from third parties in the performance of everyday activities [14]. For some people, it becomes necessary to use care facilities. In turn, institutionalization in older people deepens the process of loss of adaptive abilities that occurs with age, and resistance to adversity, as does the diagnosis of mental disorders. A relationship was also found between the decline in adaptive abilities of psychiatric patients staying in nursing homes, especially in psychotic and mentally disabled patients [15]. One of the confirmed actions reducing the risk of institutionalization of older people is the introduction of regular adapted physical activity (APA) that improves physical and mental functions [16]. APA should be treated as a prevention against loss of independence, especially since the vast majority of older people do not meet the minimum requirements for the level of physical activity necessary to maintain health, leading to the premature onset of frailty [17]. 

In the case of evaluating the functioning of the elderly, many studies have shown a significant impact of age on the daily functioning of seniors in terms of complex everyday activities, stating a decrease in functional efficiency with increasing age [18,19,20,21]. Researchers’ opinions on the relationship between gender and independence in everyday activities are divided, in some of the studies women were more often disabled in these activities than men [19,22], and others did not find any significant differences between the sexes [18,23]. In terms of instrumental activities of daily living (IADL), financial capacity is of great importance for older people. It covers a variety of activities and specific skills, including executive skills (such as coin/currency arithmetic, paying bills, etc.) and decision-making and judgment skills. It is clearly related to general cognitive and neuropsychological efficiency [24].

The coexistence of depressive symptoms reduces the financial capacity of both older adult men and women with mild Alzheimer’s disease [25], vascular dementia [26], as well as older adults with depression without a diagnosis of neurocognitive disorders [27]. Cognitive dysfunction combined with depressive disorders also significantly impairs the financial capacity of patients [28]. Financial deficits in patients with mixed dementia and coexisting depression require a thorough neuropsychological assessment to prevent financial exploitation [29].

The relationship between failure in daily activities and the occurrence of depressive disorders has been confirmed in numerous studies. People with disabilities in basic and complex activities and with reduced efficiency of cognitive functions, more dependent on others and with greater deficits in self-care were more likely to be depressed. Research results confirm that older people with greater functional impairment were more likely to experience depressive symptoms, similarly demonstrated by other researchers [18,30,31,32].

Elderly depression is a serious and growing social problem, although in practice it seems to be diagnosed too rarely. It increases mortality rates, is associated with the unfavorable course of somatic diseases, intensifies disability and leads to loss of independence, affecting the quality of life of patients and their relatives. This disorder is a challenge at the stage of diagnosis due to the masking of depressive symptoms by other psychological and health problems occurring in this period of life [33]. The diagnosis of depression in the elderly is a challenge for researchers. Ailments reported by the patient or his family may be both symptoms of dementia and depression. Dementia may also intensify the occurrence of depression and be its mask [34]. Frequent coexistence of depressive disorders and somatic diseases may delay the entire diagnostic process and accelerate the evolution of both disorders [35]. When examining the prevalence of depressive disorders in Poland in the WOBASZ study, symptoms of depression were confirmed in over 25% of the examined population on the basis of the Beck Depression Scale [36]. On the other hand, in the PolSenior study, in which the Geriatric Depression Rating Scale (GDS) was used, the incidence of age-related depressive disorders ranged from 20% to 33%, depending on the age of the respondents [31].

In another study, the prevalence of major depression in the elderly population ranged from 30 to 45%, with 10–12% of these patients admitted to an intensive care unit and 12–14% to a nursing home [37]. Some researchers confirm the thesis that age is an important factor influencing the increased risk of depression [30,31,38,39]. Others have not observed such a relationship [18,40].

Depressive symptoms may be part of the clinical picture of dementia, which has led to a debate about the direction of causality: whether depression is a prodromal symptom or an independent risk factor for dementia. Cohort studies with longer follow-ups indicate a relationship between the number of depressive episodes and the risk of dementia, which confirms the thesis that depression is a risk factor for dementia [41]. The incidence of cognitive decline in combination with depression in the elderly is high and both factors influence each other [42]. It has been documented that cognitive impairment occurs in 85–94% of cases of an acute depressive episode and 39–44% of the time after a depressive episode has subsided [43]. On the other hand, neurodegenerative dementia is accompanied by depression in 15–23% of cases [44].

Few studies report differences between men and women in the observed relationship between depression and dementia [45]. Current knowledge and conducted scientific research prove that the prognosis is equally good for the treatment of depressive syndrome, regardless of age [46]. However, a big problem is the lack of screening tests, more than 82% of the surveyed patients declared that primary care physicians omit the issue of mental well-being during the general examination. Taking into account the prevalence of depression in the elderly and the risks it entails, this situation requires rapid changes [47]. The lack of a systemic examination of the state of cognitive, physical and mental functions and the ability to function independently in order to implement prompt treatment and prevention of further impairments was described by many Polish researchers [31,33,36].

In Poland, from January 2022, the National Health Fund (NFZ) introduced a mandatory assessment of patients aged 60+, referred to hospitalization [48]. Until now, it was only applicable to patients in geriatric wards. The healthcare provider is required to document the geriatric assessment performed by a geriatric physician or geriatric consultative team. On the day of admission, the doctor performs a screening assessment using the Vulnerable Elders-13 Survey (VES-13). If the patient scores at least 3 points, he/she is qualified for the Comprehensive Geriatric Assessment (CGA). It is a widely recognized and repeatedly validated research tool used to assess the health and functioning of the elderly. It consists of questionnaires assessing the severity of depression, independence in everyday life, physical condition and cognitive abilities. The results obtained in the questionnaires, together with the assessment of the patient’s health condition and the degree of nutrition, are assessed by a geriatrician, who directs patients in need to specialist geriatric care.

Currently, the Polish medical literature lacks current reports on the results of the CGA, which has been in force since January 2022, therefore the aim of the study was to analyze the incidence of depressive disorders and their impact on the cognitive and physical functioning of the elderly and independence in the field of daily activities based on the results of the CGA conducted in patients of a multi-profile hospital.

## 2. Materials and Methods

### 2.1. Participants

The study involved 852 patients aged 60 years at the Central Teaching Hospital of the Medical University of Lodz. The study was conducted between September 2022 and June 2023. The inclusion criteria for the participants were: (1) age of at least 60 years; (2) a score of at least 3 or more points in the Vulnerable Elders-13 Survey (VES-13) scale performed during admission to the hospital. The VES-13 scale is used to assess the health of the older people. It aims to identify people at risk of a sudden deterioration in health or death in the course of age-related diseases. The subjects were patients of the following clinics: cardiology, cardio surgery, electrocardiology, internal medicine, diabetology, endocrinology, surgery, nephrology, gastrology and rehabilitation. These were people admitted on a scheduled basis for diagnostic tests, surgical and conservative treatment or in an emergency due to a sudden deterioration—health status.

### 2.2. Desing and Procedure

The participants who were qualified for the study were examined using four different tools: the Geriatric Depression Scale (GDS), Instrumental Activities of Daily Living (IADL), The Timed Up and Go (TUG) and Schulman’s Clock-Drawing Test. The Comprehensive Geriatric Assessment was carried out on the second or third day of hospitalization, which in the case of patients after surgical procedures, invasive diagnostics or in the state of persistent symptoms of health deterioration sometimes made it impossible to conduct a full examination. The results of the questionnaires together with diagnostic tests and the assessment of the patient’s general health were the basis for the geriatrician to qualify the patient for permanent specialist geriatric care.

The study was approved by the Institutional Review Board, approval Ref: RNN/200/23/KE.

### 2.3. Instruments

The Geriatric Depression Rating Scale (GDS) according to Yesavage is used for screening depressive disorders in older people. The person can get a maximum of 15 points by answering the questions. The interpretation of the results is as follows: a score between 0–5 means no depression; a score between 6–10 means moderate depression and a score between 11–15 means severe depression. Based on the current literature, many studies reported that the mean internal consistency coefficient was 85 and the test-retest coefficient was 83. [49].

Activities of Daily Living (IADL) questionnaire is used to assess the level of physical disability in older people. The person is asked to define his/her abilities in the scope of 8 daily activities. For every activity performed independently, the patient receives 1 point. The interpretation of the results is as follows: the total score between 5–6 points means no disability, a score between 3–4 points means moderate disability and a score between 0–2 points means severe disability. Based on the study on the Polish population, the reliability coefficients of the scale measured with the internal consistency method and test-retest were ≥0.80 [50].

Test Time Up and Go (TUG) is used to assess the physical fitness of the elderly. The course of the test is as follows: a person gets up from the chair on time, walks a distance of 3 m, turns around, returns and sits on the chair as quickly as possible. The interpretation of the results is as follows: time of the covered distance below 10 s means a correct functional efficiency; time between 10–19 s means that the person can go outside on his/her own, does not need any walking aids, is independent in most activities of daily living, but has a low risk of falls; time over 19 s means that the person has a significantly limited functional capacity, cannot go outside on his/her own and has a high risk of falls. Here, the walking aids are recommended. 

Clock Drawing Test (CDT) allows for quick detection of cognitive and executive function deterioration. The patient draws a clock face on a blank sheet of paper, marking the time “11.10”. In our study the person gets a point from 1 to 5 according to the correctness of the task: 1 point means a correctly drawn clock; 2 points mean minor visual-spatial errors; 3 points mean a wrong designation 10 after 11 with a generally good visuo-spatial organization; 4 points mean the visuospatial difficulties, correct marking of 10 after 11 is impossible; 5 points mean inability to fill the clock face in a clock-like manner and 6 points refers to ‘no reasonable attempt of a clock’. There are several different well-validated scales to interpret the result of CDT. However, there is no consensus about which of them is the most reliable. The methods by Shulman et al. demonstrated the best diagnostic accuracy, therefore the results were analyzed according to the Shulman protocol [51].

### 2.4. Statistical Analysis

Statistical analyses were performed using Statistical version 13.1 software (StatSoft, Tulsa, OK, USA). The Shapiro–Wilk test was used to check whether the data had a normal distribution. Normally distributed measurement data were expressed as mean ± standard deviation (SD) and the enumeration data were expressed in numbers and percentages. The Chi^2^ test was performed to analyze the differences between and inside the groups. Multivariance ANOVA was used for comparison of measurement data between groups, and post-doc least significant difference (LSD) was used for comparison of data between and within groups. Spearman correlation test was used to analyze the relationship between GDS, IADL, TUG and CDT. Significant differences were accepted for all analyses at the level of *p* < 0.05.

## 3. Results

### 3.1. Characteristics of the Study Group

Half of the study participants, both females and males, did not have any depressive disorders and were not disabled. However, almost 30% of the female and male participants were characterized by a moderate level of depressive disorders or severe disability. Most of the participants had a correct functional efficiency, but if not, their physical fitness was characterized by a low risk of falls. One-third of the participants, both males and females, were not able to obtain satisfying results from CDT (Table 1). Based on the mean values of GDS, IADL, TUG and CDT, there were statistically significant differences between females and males in the results. Generally, females had worse results in GDS and TUG tests than males, but slightly better in CDT (Table 2).

Analysing the IADL results, it was observed that half of the study participants had problems with doing shopping, preparing meals, cleaning or washing, both males and females. In opposition to males, the study females more often were noticed to have problems with different forms of transport (Table 3).

### 3.2. Spearman Correlation Test

Spearman’s correlation test identified a relationship between the results of IADL, TUG, CDT and GDS. A strong negative relationship statistically significant (*p* < 0.05) was observed between GDS related to depressive disorders and IADL results related to disability level. Additionally, a strong positive relationship statistically significant (*p* < 0.05) was observed between GDS and CDT related to cognitive disorders. There was observed a negative small relationship between GDS and TUG results related to physical fitness, but with no statistical significance. In contrast, there was a small, but positive relationship between IADL and TUG results A moderate negative relationship was also observed between IADL and CDT with *p* < 0.05 (Table 4). Similar correlations were observed in males and females (Table 5 and Table 6). 

The Spearman correlation test was provided to identify a relationship between the results of GDS and instrumental activities assessed by IADL. Generally, a negative relationship was observed between GDS related to depressive disorders and every IADL activity. A statistically significant relationship was observed especially among the activities related to using a phone, medications and money. In females, also a negative statistically significant relationship was observed between GDS results and the activity related to using transport (Table 7).

### 3.3. Relationship between the Level of Depressive Disorders and Cognitive and Physical Function

**Figure 1:**
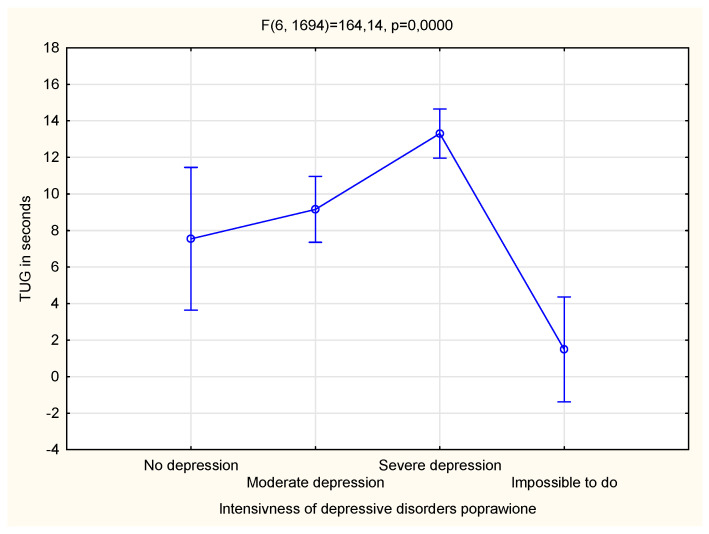
The relationship between the level of depressive disorders and physical fitness.

**Figure 2:**
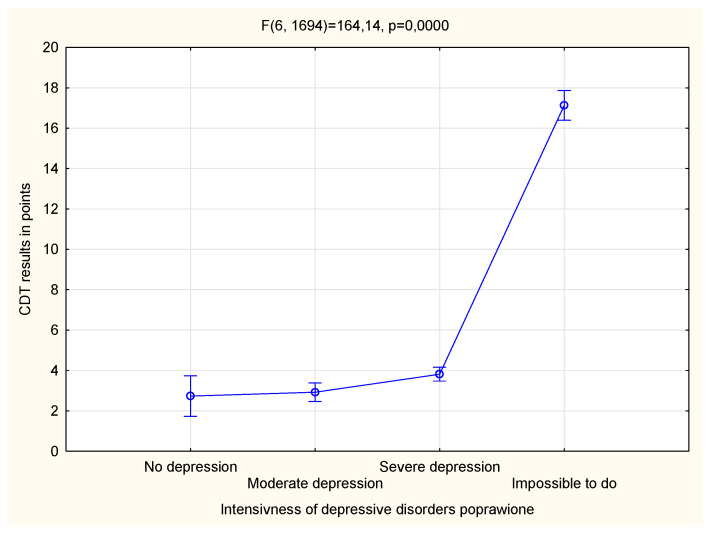
The relationship between the level of depressive disorders and cognitive impairment.

Figure 1 and Figure 2 present the association between the TUG test related to physical fitness and the CDT test related to cognitive function. It can be observed that the results of the tests depended significantly on the severity of the diagnosed depressive disorders (Figure 1 and Figure 2).

No relationship between the GDS test results and sex was observed. There was an observed relationship between GDS test results, TUG and CDT test results, but it was not statistically significant. There was also observed the statistically significant relationship between GUS and IDAL results with *p* < 0.001 and the interaction between GDS, IDAL and sex with *p* < 0.05 (Table 8).

## 4. Discussion

Dementia is a neurodegenerative disease that results mostly in a decline in cognition abilities due to neuron death affecting the independent functioning of older people [52]. The primary risk factor for dementia development is age. Research shows that more than 50% of 90-year-olds have a dementia disorder and according to WHO, there are nearly 10 million new cases every year. It is also estimated that the total number of people with dementia will reach 82 million in 2030 and 152 million in 2050 [53]. WHO recognizes dementia as a public health priority [54]. Currently, there is no reliable test or sufficiently developed diagnostic tools for the early identification of dementia or screening of its first symptoms. Although there are different scales or indices that can be used to measure the severity of symptoms, such as cognitive deterioration, functional decline and behavioral changes which can be the predictors of dementia development, studies are lacking that have combined different types of testing and showing which approaches are most effective [52,55]. Apart from dementia, depression is one of the most challenging mental health problems in late life which is even more common among older people than in adolescents [56]. There were identified several risk factors for dementia and among them, depression seems to have an important role [57]. Therefore, the present study was to analyze the frequency of depressive disorders among older people and notice its relationship with cognitive and physical impairment in older people. Previous studies have indicated that it is of great importance to examine the association between cognitive impairment in older patients with depressive disorder for early diagnosis and timely treatment intervention [58].

The results of the study showed that over 35% of the participants were diagnosed with depressive symptoms. Moreover, most of them were characterized by the moderate level of depressive symptoms and it was similar among males and females. It is in line with the meta-analysis in which it was found that over a third of elderly populations globally were diagnosed with depression symptoms [59]. In turn, in the PolSenior study, in which the Geriatric Depression Rating Scale (GDS) was used, it was noted that the prevalence of depressive disorders increases with age (20% in the 55–59 age group; 25% in the 65–79 age group; 33% in the age group of 80 and more years) [31]. In the study of the prevalence of depressive symptoms in 202 people aged 65 and over in treatment and care facilities in the Bielsko district in Poland, the mean values of the GDS test for the entire study population were 11.7 ± 6.5 points. The absence of a depressive mood was found in 45.6% of the subjects, mild symptoms of depression in 43.6%, and severe depression in 10.9% of the subjects. A statistically significant positive relationship between the GDS results and the sex of the subjects was demonstrated [60]. In turn, in a study of 151 people over 65 who are patients of the Geriatrics Clinic of Collegium Medi-cum im. Ludwik Rydygiera in Bydgoszcz, using the Geriatric Depression Rating Scale in the extended version, depressive symptoms were found in 41.72% of the respondents (of which 77.78% were mild depression). Of these, 5 people were patients with depression diagnosed earlier—treated during the study, while the rest were cases of newly diagnosed depressive disorders. A relationship between the occurrence of depressive symptoms and the advancing age of the subjects and the female gender was found [47].

In China’s population study, it was noticed that the depression tendency in the elderly was associated with the cognitive function change trajectory, so attention should be paid to the mental health of older people to prevent the development of dementia [61]. In the present study, one-third of the participants, both males and females, were not able to obtain satisfying results of CDT which means that their cognitive function was impaired. Unfortunately, it is evidenced that people with cognitive impairment, especially the mild level of CI, are 3 to 4 times more likely to develop dementia in comparison to those with normal cognitive function [62].

According to the test results, about one-third of the participants were noticed to have severe impairment of physical fitness with a high risk of falls. However, almost one-fifth of the study participants were characterized with a physical impairment which enabled them to go outside on his/her own with no walking aids, they felt independent in most activities of daily living and had a low risk of falls. The rest of the participants had a correct functional efficiency. Reppermund S. et al. study confirmed no relationship between current depression with functional abilities, although it was observed with poorer cognitive performance [63]. In contrast, in the cross-sectional study among elder people aged over 65 years and with diagnosed MCI or dementia, there was observed a significant relationship between physical fitness and cognition, except the components such as flexibility [64]. 

There were statistically significant differences between females and males in the results. Generally, females had worse results related to depressive disorders and those results characterized physical fitness. However, their cognitive function seemed to be slightly better than males. Evidence shows that there are differences between the sexes in the rate of cognitive decline with aging [65], which is explained by estrogens which can be helpful in maintaining cognitive function, especially in women who are current users of hormone therapy [66]. 

Depression disorders were related to greater impairment of cognitive function of the study participants which has been confirmed in previous research [67]. In Bellou et al. study it was reported that over half of patients who were diagnosed with depressive disorder also met the diagnosis of cognitive dysfunction [68]. In the present study, depressive disorders had an impact on the activities of daily living of the participants and the level of perceived physical disability. Moreover, in the present study, there was observed a relationship between the severity of the diagnosed depressive disorders and the physical fitness and cognitive function test results. Some other studies indicated also the inverse relationship, namely that physical function may influence the course of depressive disorders [69]. 

To sum up, the study showed that depressive disorders are frequent among the older population and affect 1 in 3 people. The cognitive impairment of older people and physical fitness can be associated with the degree of severity of depressive disorders. There is a need to examine if effective treatment of depressive disorders has any effect in reducing the prevalence of dementia. In addition, it is important to be vigilant with the clinicians for depression development indicating also the possible development of dementia in older people, and therefore carefully following up these individuals for future cognitive impairment. 

There are several strengths of the present study, including large sample size, simultaneous use of several tests enabling a multi-aspect assessment of the patient and the use of methods which are internationally recognized standardized tools. It is also worth to underline that the tests for older people were conducted by the same researcher, so the results were not influenced by the subjective assessment of different people. However, some limitations should be acknowledged. Firstly, the study population was only from one medical center in the country which may affect the incidence of depression. The incidence of the phenomenon may vary depending on the region of the country, so the results cannot be generalized to the whole elderly people and treated with caution. Further, multi-centered studies would be recommended. Secondly, we did not make a direct analysis of depression, but it can be carried out by a professional psychiatrist and not by a self-reported questionnaire. Our study also does not take into account the time of occurrence of the first symptoms of depression, which can help to differentiate subtypes of depression in old age. In addition, the results could be supplemented with additional morphological and biochemical tests or other information related to the health status of the older people which could be cofounders influencing the study results.

## 5. Conclusions

Observing the first signs of depression among older people may be considered an indication for further diagnosis of dementia. Therefore, mandatory screening of older people for dementia should be introduced, and extended by tools diagnosing depressive disorders. This will allow for early diagnosis and appropriate preventive measures to slow down the development of the disease and maintain independence and good quality of life for older people as long as possible.

## Figures and Tables

**Table 1:** Characteristics of the study group.

	ALL	MALES	FEMALES
	N (%)		
GDS			
No depression	452 (52.99) *	177 (57.65) *	275 (50.37) *
Moderate depression	250 (29.31) *	84 (27.36) *	166 (30.40) *
Severe depression	53 (6.21)	15 (4.88)	38 (6.96)
Impossible to do	98 (11.49)	31 (10.10)	67 (12.27)
IADL			
Severe disability	255 (29.89) *	88 (28.66)	167 (30.59)
Moderate disability	146 (17.12)	49 (15.96)	97 (17.76)
No disability	452 (52.99) *	170 (55.37)	282 (51.65)
TUG			
Correct functional efficiency	436 (51.11) *	163 (53.09) *	273 (50.00) *
Low risk of falls	281 (32.94) *	105 (34.20) *	176 (32.23) *
High risk of fall	136 (15.94)	39 (12.70)	97 (17.76)
CDT			
Correct drawing	186 (21.80)	53 (17.26)	133 (24.36)
Minor visual-spatial errors	86 (10.08)	17 (5.54)	69 (12.64)
Wrong designation 10 after 11	128 (15.01)	38 (12.38)	90 (16.48)
Visuospatial difficulties	111 (13.01	53 (17.26)	58 (10.62)
Inability to draw a clock	258 (30.25) *	119 (38.76) *	139 (25.46) *
Impossible to do	83 (9.73)	27 (8.77)	57 (10.44)

* *p* < 0.05.

**Table 2:** The mean values of the results obtained by the study participants.

	ALL (N = 853)	MALES (N = 307)	FEMALES (N = 546)	t	df	*p*
	Mean (±SD)					
Geriatric Depression Scale (GDS)	7.02 ± 6.18	6.32 ± 6.03	7.41 ± 6.24	−2.47	853	0.014 *
Instrumental Activities of Daily Living (IADL)	4.53 ± 2.98	4.75 ± 3.01	4.41 ± 2.96	1.57	853	0.116
The Timed Up And Go (TUG)	10.36 ± 14.97	9.01 ± 10.32	11.13 ± 17.00	−1.98	853	0.048 *
Clock-Drawing Test (CDT)	5.02 ± 5.74	5.16 ± 5.34	4.94 ± 5.96	−2.17	853	0.030 *

* *p* < 0.05.

**Table 3:** The analysis per item of the IADL scale in the study sample.

	ALL (N = 853)		MALES (N = 307)		FEMALES (N = 546)	
	N (%)					
	Yes	No	Yes	No	Yes	No
IADL 1 ACTIVITY: USING A PHONE	716 (83.94)	137 (16.06) *	258 (84.04)	49 (15.96) *	458 (83.88)	88 (16.12) *
IADL 2 ACTIVITY: DOING SHOPPING	350 (41.03)	503 (58.97)	156 (50.81)	151 (49.18)	194 (35.53)	352 (64.47) *
IADL 3 ACTIVITY: PREPARING MEALS	446 (52.29)	407 (47.71)	154 (50.16)	153 (48.84)	292 (53.48)	254 (46.52)
IADL 4 ACTIVITY: CLEANING	405 (47.48)	448 (52.52)	153 (49.84)	154 (50.16)	256 (46.15)	290 (53.85)
IADL 5 ACTIVITY: WASHING	456 (53.46)	397 (46.54)	151 (49.18)	156 (50.81)	305 (55.86)	241 (44.14)
IADL 6 ACTIVITY: USING DIFFERENT FORMS OF TRANSPORT	303 (35.52)	550 (64.48) *	144 (46.90)	163 (53.09)	159 (29.12)	387 (70.88) *
IADL 7 ACTIVITY: USING MEDICATIONS	574 (67.29)	279 (32.71) *	206 (67.10)	101 (32.90) *	368 (67.40)	178 (32.60) *
IADL 8 ACTIVITY: USING MONEY	621 (72.80)	232 (27.20) *	235 (76.55)	72 (23.45) *	386 (70.70)	160 (29.30) *

* *p* < 0.05.

**Table 4:** Correlations between the results of GDS, IADL, TUG and CDT test results in the whole study group.

	GDS	IADL	TUG	CDT
GDS	1.00	−0.61 *	−0.24	0.74 *
IADL	−0.61 *	1.00	0.28	−0.54 *
TUG	−0.24	0.28	1.00	−0.25
CDT	0.74 *	−0.54 *	−0.25	1.00

* *p* < 0.05.

**Table 5:** Correlations between the results of GDS, IADL, TUG and CDT test results in males.

	GDS	IADL	TUG	CDT
GDS	1.00	−0.62 *	−0.34	0.75 *
IADL	−0.62 *	1.00	0.34	−0.53 *
TUG	−0.34	0.34	1.00	−0.28
CDT	0.75 *	−0.53 *	−0.28	1.00

* *p* < 0.05.

**Table 6:** Correlations between the results of GDS, IADL, TUG and CDT test results in the whole study group in females.

	GDS	IADL	TUG	CDT
GDS	1.00	−0.61 *	−0.22	0.73 *
IADL	−0.61 *	1.00	0.27	−0.54 *
TUG	−0.22	0.27	1.00	−0.25
CDT	0.73 *	−0.54 *	−0.25	1.00

* *p* < 0.05.

**Table 7:** The analysis of the relationship between the depressive symptomatology measured and instrumental activities.

	GDS		
	ALL	MALES	FEMALES
IADL 1	−0.46 *	−0.48 *	−0.45 *
IADL 2	−0.42	−0.45	−0.38 *
IADL 3	−0.40	−0.43	−0.40
IADL 4	−0.41	−0.42	−0.40
IADL 5	−0.41	−0.42	−0.43
IADL 6	−0.42 *	−0.47	−0.37 *
IADL 7	−0.47 *	−0.46 *	−0.49 *
IADL 8	−0.51 *	−0.53 *	−0.49 *

* *p* < 0.05.

**Table 8:** Multivariate analysis of variance (MANOVA) comparing the results of GDS with other variables.

	Value	F	df	Mean Square	*p*
SEX	0.979	4.32	4	841.000	0.002 *
GDS	0.293	109.38	12	2225.368	0.001 *
GDS × SEX	0.993	0.47	12	2225.368	0.934
GDS × TUG	0.975	1.12	18	1624	0.320
GDS × IDAL	0.922	3.82	18	2370.707	0.001 *
GDS × CDT	0.975	1.12	18	1624	0.319
GDS × SEX × TUG	0.974	1.17	18	1624	0.280
GDS × SEX × IADL	0.974	1.78	12	2190.974	0.046 *
GDS × SEX × CDT	0.974	1.17	18	1624	0.279

* *p* < 0.05.

## Data Availability

The data presented in this study are available on request from the corresponding author.
